# Fruit and vegetable consumption in Europe according to gender, educational attainment and regional affiliation—A cross-sectional study in 21 European countries

**DOI:** 10.1371/journal.pone.0232521

**Published:** 2020-05-13

**Authors:** Tonje Holte Stea, Oda Nordheim, Elling Bere, Per Stornes, Terje Andreas Eikemo

**Affiliations:** 1 Department of Health and Nursing Science, University of Agder, Kristiansand, Norway; 2 Department of Sociology and Political Science, Centre for Global Health Inequalities Research (CHAIN), Norwegian University of Science and Technology (NTNU), Trondheim, Norway; 3 Department of Health and Inequalities & Centre for Evaluation of Public Health Measures, Norwegian Institute of Public Health, Oslo, Norway; 4 Department of Sport Science and Physical Education, University of Agder, Kristiansand, Norway; University of Florida, UNITED STATES

## Abstract

**Objective:**

The purpose of the present study was to examine fruit and vegetable consumption according to gender, educational attainment and regional affiliation in Europe.

**Design:**

Cross-sectional study.

**Setting:**

21 European countries.

**Participants:**

37 672 adults participating in the 7^th^ round of the European Social Survey.

**Main outcome measures:**

Fruit and vegetable consumption was measured using two single frequency questions. Responses were dichotomized into low (<once a day) and high (≥once a day) consumption. The association between consumption of fruit and vegetables and gender, educational level, regional affiliation was examined using logistic regression analyses.

**Results:**

Overall, females showed increased odds of consuming fruit (OR 1.71 (95%CI:1.62, 1.79) and vegetable (1.59 (1.51, 1.67)) compared to males and high educated participants showed increased odds of consuming fruit (1.53 (1.43, 1.63)) and vegetables (1.86 (1.74, 2.00)) compared to low educated participants. Our results also showed that participants living in Eastern Europe had the lowest odds of consuming fruit and vegetables, whereas participants from Southern- and Northern Europe had the highest odds of consuming fruit and vegetables, respectively. Results from interaction analyses confirmed the positive association between fruit and vegetable consumption and educational level, although for some European regions, decreased odds of fruit and vegetables was observed among medium educated participants compared to those with low education.

**Conclusions:**

Overall, the present study showed that being female and having a high education were associated with increased consumption of fruit and vegetables. However, the direction and strength of these relationships depends on regional affiliations.

## Introduction

Studies have confirmed that low consumption of fruit, vegetables and fruit and vegetables combined, is associated with increased risk of cardiovascular disease, type 2 diabetes, cancer, and all-cause mortality.[1,2,3] In addition to positive health impacts, a shift towards healthier plant-based diets will most likely have important environmental impacts by reducing the environmental footprint.[4] Thus, increasing fruit and vegetable consumption is an important component of a shift towards both healthier and more sustainable diets.

Organized health promotion and disease prevention efforts in most European countries have focused specifically on initiatives to increase the consumption of fruit and vegetables among different groups.[5,6] The well-known 5/6-a-day campaign was originally initiated by the National Cancer Institute in the U.S in the 1990s, and later implemented in several European countries, but despite this and other health promoting initiatives, less than half of the countries of the WHO consume fruit and vegetables according to the WHO recommendations.[5]

Educational attainment has been identified as one of the most important predictors of fruit and vegetable consumption and has also been positively associated with diet quality, diversity and higher odds to comply with national recommendations.[7,8,9] Studies have further shown a higher consumption of fruit and vegetables among women compared to men, and that gender differences in the relationship between socioeconomic status (SES)/educational level and fruit and vegetable consumption exists.[10–12]

Although most European studies have reported a graded relationship between educational attainment and fruit and vegetable consumption, this is not a consistent finding. [10,13–16] Some studies have shown that in countries and regions characterized by high availability and consumption of fruit and vegetables, those with low education tend to have a higher consumption of these food items compared to those with high education.[17,18] Inconsistent findings may partly be explained by a general lack of comparable estimates due to different study designs; most previously published studies have focused on single countries,[15,19–21] whereas systematic reviews have based their conclusion on studies using different designs and modes of data collection.[16,18,22]

The health module of the 7th round of the European Social Survey provides us with a unique opportunity to overcome these limitations with reliable and comparable estimates. Thus, the aim of the present study was to examine how the consumption of fruit and vegetables varies according to gender, educational attainment and regional affiliation in a large representative sample of the European adult population.

## Materials and methods

### Design

The data was collected in 2014 as part of round 7 of the European Social Survey (ESS), in face-to-face interviews, using a standardized procedure, and designed to be representative of all persons aged 15 and over, resident within private households in each country. Written informed consent was obtained from all participants prior to data collection. The ESS ERIC Research Ethics Committee **(**REC) was established in 2015, and the present study was approved in accordance with the ESS ERIC Statutes (Article 23.3). The ESS ERIC subscribes to the Declaration on Professional Ethics of the International Statistical Institute, which can be downloaded from https://www.isi-web.org/index.php/activities/professional-ethics/isi-declaration.

The total sample included 37 762 respondents. More information regarding the European Social Survey has been previously published elsewhere.[23] The following 21 countries were included: Austria (response rate: 52%), Belgium (57%), Czech Republic (68%), Denmark (52%), Estonia (60%), Finland (63%), France (51%), Germany (31%), Hungary (53%), Ireland (61%), Israel (74%), Lithuania (69%), Netherlands (59%), Norway (54%), Poland (66%), Portugal (43%), Slovenia (52%), Spain (68%), Sweden (50%), Switzerland (53%), and United Kingdom (44%).

The data utilized for analyses in the present study was based on a self-report questionnaire which focused on attitudes, beliefs and behaviour patterns. Fruit consumption was measured by asking respondents ‘How often do you eat fruit, excluding drinking juice? Frozen fruit should be included’. Vegetable consumption was measured by asking ‘How often do you eat vegetables or salad, excluding potatoes? Frozen vegetables should be included’. Response categories for both fruit and vegetable consumption were: ‘1. Three times or more a day; 2. Twice a day; 3. Once a day; 4. Less than once a day but at least 4 times a week; 5. Less than 4 times a week but at least once a week; 6. Less than once a week; 7. Never. A consumption of once a day or more was acknowledged as an acceptable frequency of consuming these food items. Thus, consumption of both fruit and vegetables were further dichotomized into once a day or more (category 1–3; high consumption) or less than once a day (category 4–7; low consumption as reference category). The survey did not provide data on quantity and variety of fruit and vegetable consumption.

The variable capturing age was coded into 5-year increments, from 15 upwards and capped at 79. Gender was coded as a dummy variable (reference females). Educational attainment was coded into three categories where no education, primary education and lower secondary education was coded as ‘low education’, upper secondary education was coded as ‘medium education’ and tertiary education was coded as ‘high education’. Dummy variables were also created to capture regional affiliation. The countries coded as Northern European include Denmark, Estonia, Finland, the UK, Ireland, Lithuania, Norway and Sweden. The countries coded as Western European include Austria, Belgium, Switzerland, Germany, France and the Netherlands. Finally, countries coded as Eastern European include Czech Republic, Hungary and Poland, while Portugal, Slovenia, Spain and Italy were coded as South Europe.

### Statistical analysis

Statistical analyses were carried out using Stata 16. We have presented descriptive statistics, including age, gender, education and fruit and vegetable consumption for each of the 21 European countries as well as mean values for the pooled sample.

Further, fruit and vegetable consumption were analysed separately, using logistic regression analyses to estimate odds ratios for high frequency of fruit and vegetable consumption according to gender, education and regional affiliation. The ESS post-stratification weight was applied in all analyses. In the first part of the analyses, we examined odds ratios for high frequency of fruit and vegetable consumption according to gender, education and regional affiliation. Based on the results from these analyses, we further examined how interactions between regional affiliation and education affect frequency of fruit and vegetable consumption among men and women, respectively. Thus, we used regression analyses stratified by gender to examine how FV consumption varied among participants from different European regions according to educational level using low educated Eastern European males or females as reference groups, respectively. All regression models were adjusted for age and the likelihood ratio test confirmed that the interactions between region and education were statistically significant contributors to the models focusing on both fruit and vegetable consumption (p<0.001 for both). We also conducted a chow test which confirmed that education and region have a significantly different impact on consumption frequency among women compared to men, related to both fruit and vegetables (p<0.001 for both).

## Results

Descriptive results are presented in Table 1. The total sample was 37 672, with single country samples ranging from 1161 (Slovenia) to 2891 (Germany). For the pooled sample, the mean age was 47 years, and there was a slightly higher proportion of females (52.6%). Mean age for the single countries varied from 45 (Belgium) to 50 (Portugal), and the proportion of women from 46% (Norway) to 61% (Lithuania). Educational level was low for 25%, medium for 51% and high for 23% of the pooled sample.

**Table 1:** Age, gender, education and fruit and vegetable consumption in the European Social Survey round 7 (n = 37 672).

Country	N	Mean age	Females (%)	Education (%)			Fruit cons. (%)	Veg cons. (%)
				*Low*	*Med*.	*High*	*High*^1^	*High*^1^
AT	1 705	48	52	20	65	15	64	58
BE	1 664	45	49	29	36	35	65	85
CH	1 460	46	50	22	48	30	73	83
CZ	2 074	46	53	12	72	15	53	46
DE	2 891	49	49	12	52	36	71	67
DK	1 426	46	48	22	34	43	72	71
EE	1 911	48	58	14	52	33	68	70
ES	1 788	46	48	53	22	24	75	56
FI	1 942	49	50	22	39	39	70	77
FR	1 781	47	52	22	44	34	67	76
GB	2 015	50	55	33	25	40	72	78
HU	1 622	49	57	19	61	19	42	36
IE	2 226	48	54	36	32	30	73	81
IL	2 329	46	55	15	44	40	70	79
LT	2 134	49	61	22	44	33	54	68
NL	1 805	49	55	35	30	34	70	78
NO	1 378	45	46	18	39	43	71	78
PL	1 534	46	54	43	37	20	69	71
PT	1 165	50	54	62	19	18	84	78
SE	1 661	47	50	18	45	36	62	78
SI	1 161	48	54	21	54	25	80	81

Country abbreviations: HU-Hungary, NL-Netherlands, AT-Austria, CZ-Czech Republic, DE-Germany, ES-Spain, PL-Poland, EE-Estonia, BE-Belgium, SI-Slovenia, NO-Norway, LT-Lithuania, IE-Ireland, DK-Denmark, CH-Switzerland, SE-Sweden, FI-Finland, GB-United Kingdom, IL-Israel, FR-France, PT-Portugal.

^1^At least one fruit or vegetable per day

Regression analyses for the pooled sample adjusted for age (Table 2) showed increased odds of consuming fruit and vegetables among females (OR 1.71 (1.62, 1.79) and OR 1.59 (1.51, 1.67), respectively) and among high educated participant (OR 1.53 (1.43, 1.63) and OR 1.86 (1.74, 2.00, respectively) compared to males and low educated participant. Further results revealed increased odds of consuming fruit and vegetables among participants from Northern Europe (OR 1.66 (1.54, 1.78) and OR 2.79 (2.59, 3.00), respectively), Western Europe (OR 1.73 (1.60, 1.86) and OR 2.58 (2.39, 2.78), respectively) and Southern Europe (OR 2.51 (2.29, 2.74) and OR 2.53 (2.32, 2.76), respectively) compared to those from Eastern Europe (reference category).

**Table 2:** Consumption frequency of fruit and vegetables according to gender and educational level (n = 37 672).

	High fruit consumption	High vegetable consumption
VARIABLES	OR (95% CI)	OR (95% CI)
**Gender**		
Male (ref)	1.00	1.00
Female	1.71 (1.62, 1.79)***	1.59 (1.51, 1.67)***
**Education**		
Low (ref)	1.00	1.00
Medium	1.04 (0.98, 1.10)	1.16 (1.09, 1.24)***
High	1.53 (1.43, 1.63)***	1.86 (1.74, 2.00)***
**Regional affiliation**		
Eastern Europe (ref)	1.00	1.00
Northern Europe	1.66 (1.54, 1.78)***	2.79 (2.59, 3.00)***
Western Europe	1.73 (1.60, 1.86)***	2.58 (2.39, 2.78)***
Southern Europe	2.51 (2.29, 2.74)***	2.53 (2.32, 2.76)***

Reference groups are marked with OR = 1.00

All analyses were adjusted for age

* p<0.1, ** p<0.05

*** p<0.01

Analyses presented in Table 3 show how the interactions between regional affiliation and education was associated with fruit and vegetable consumption. In Eastern Europe, the results showed increased odds of consuming fruit among high educated females compared to low educated females (OR 2.16 (1.65, 2.81). Likewise, increased odds of consuming vegetables were observed among high (OR 2.06 (1.60, 2.66) educated females living in Eastern Europe.

**Table 3:** Interactions between regional affiliation and educational level and odds for high frequency of fruit and vegetable consumption among males (n = 17 845) and females (n = 19 827).

	High fruit consumption		High vegetable consumption	
VARIABLES	Males	Females	Males	Females
	OR (95% CI)	OR (95% CI)	OR (95% CI)	**OR (95% CI)**
**Eastern Europe**				
*Low education (ref)*	1.00	1.00	1.00	1.00
Medium education	0.86 (0.69, 1.06)	1.24 (1.02, 1.52)**	0.68 (0.56, 0.84)***	1.21 (1.00, 1.47)**
High education	1.12 (0.85, 1.48)	2.16 (1.65, 2.81)***	1.14 (0.87, 1.51)	2.06 (1.60, 2.66)***
**Northern Europe**				
Low education	1.46 (1.18, 1.80)***	1.53 (1.24, 1.88)***	1.66 (1.36, 2.04)***	2.68 (2.19, 3.27)***
Medium education	1.38 (1.14, 1.68)***	2.08 (1.72, 2.53)***	1.95 (1.61, 2.37)***	4.02 (3.32, 4.86)***
High education	2.26 (1.84, 2.77)***	3.52 (2.89, 4.28)***	3.53 (2.87, 4.34)***	6.41 (5.26, 7.81)***
**Western Europe**				
Low education	1.46 (1.17, 1.83)***	2.02 (1.63, 2.51)***	1.98 (1.59, 2.47)***	2.96 (2.40, 3.66)***
Medium education	1.53 (1.26, 1.87)***	2.40 (1.98, 2.92)***	1.74 (1.43, 2.11)***	3.33 (2.76, 4.03)***
High education	1.89 (1.53, 2.33)***	2.89 (2.33, 3.58)***	2.55 (2.06, 3.15)***	6.53 (5.19, 8.22)***
**Southern Europe**				
Low education	2.70 (2.14, 3.41)***	3.41 (2.69, 4.33)***	1.57 (1.26, 1.96)***	2.39 (1.93, 2.97)***
Medium education	2.13 (1.70, 2.67)***	2.54 (2.02, 3.19)***	2.45 (1.95, 3.06)***	3.42 (2.73, 4.28)***
High education	2.91 (2.26, 3.74)***	3.97 (3.11, 5.07)***	2.57 (2.01, 3.28)***	4.53 (3.57, 5.74)***

Reference groups are marked with OR = 1.00

All analyses were adjusted for age

* p<0.1

** p<0.05

*** p<0.01

Compared to low educated males from Eastern Europe (ref), the results showed increased odds of consuming fruit among low and high educated males living in Northern Europe (OR 1.46 (1.18, 1.80) and OR 2.26 (1.84, 2.77), respectively), Western Europe (OR 1.46 (1.17, 1.83) and OR 1.89 (1.53, 2.33), respectively) and Southern Europe (OR 2.70 (2.14, 3.41) and OR 2.91 (2.26, 3.74), respectively).

Likewise, increased odds of consuming fruit were observed among low and high educated females from Northern Europe (OR 1.53 (1.24, 1.88) and OR 3.52 (2.89, 4.28), respectively), Western Europe (OR 2.02 (1.63, 2.51) and OR 2.89 (2.33, 3.58), respectively) and Southern Europe (OR 3.41 (2.69, 4.33) and OR 3.97 (3.11, 5.07)) compared to low educated females from Eastern Europe (ref).

Among Eastern European participants, medium educated males showed decreased odds of vegetable consumption compared to low educated males. Furthermore, the results showed increased odds of consuming vegetables among low and high educated males from Northern Europe (OR 1.66 (1.36, 2.04) and OR 3.53 (2.87, 4.34), respectively), Western Europe (OR 1.98 (1.59, 2.47) and OR 2.55 (2.06, 3.15), respectively) and Southern Europe (OR 2.39 (1.93, 2.97) and OR 4.53 (3.57, 5.74), respectively), compared to low educated Eastern European males (ref).

For females, similar results showed increased odds of consuming vegetables among low and high educated participants from Northern Europe (OR 2.68 (2.19, 3.27) and OR 6.41 (5.26, 7.81), respectively), Western Europe (OR 2.96 (2.40, 3.66) and OR 6.53 (5.19, 8.22), respectively) and Southern Europe (OR 2.39 (1.93, 2.97) and OR 4.53 (3.57, 5.74), respectively), compared to low educated females in Eastern Europe (ref).

## Discussion

Based on the total study sample, the results showed a gender and education gradient in which being female and having high education was associated with increased consumption of fruit and vegetables. Overall, our results also showed that participants living in Eastern Europe had the lowest odds of consuming fruit and vegetables, whereas participants from Southern- and Northern European countries had the highest odds consuming of fruit and vegetables, respectively.

Although few previously published studies have compared data from many different European countries, variations in the patterns of fruit and vegetable consumption between countries and regions have been identified.[10,13–16] Similar to our findings, a systematic review has confirmed a lower consumption of fruit, but no differences in vegetable consumption between participants living in Eastern Europe and those living in other European countries.[24] As suggested by the authors, lack of differences in regional consumption of vegetables reported in the systematic review might be explained be lack of north/south weighting of health behavioral survey results.[24]

In our study, interaction analyses examining the association between regional affiliation and consumption of fruit and vegetables also revealed interesting nuances in the results; high educated Eastern European females, but not males showed significantly increased odds of consuming fruit and vegetables compared with low educated participants from this region. Medium educated males, however, showed decreased odds of consuming vegetables compared to low educated males from Eastern Europe. Furthermore, some results also indicate decreased odds of fruit and vegetables among medium educated participants compared to those with low education.

These results may partly be explained by findings from a few previously published studies, which showed that in regions characterized by high availability, high affordability and high consumption of fruit and vegetables, lower social classes tend to have a higher consumption of fruit and vegetables than higher social classes.[16,18] Roos et al[18] has also suggested that a high consumption of fruit among low educated participants may be expected in regions such as Southern Europe, with better access to cheaper and fresh commodities. Furthermore, low educated adults with high income have previously reported similar consumption of fruit and vegetables as those with intermediate or high education.[25] These findings are in line with previous studies which have identified the role of economic constraints and absolute cost of fruit and vegetables on food choices,[26,27] and that dietary costs are in the causal pathway between socioeconomic status and diet, especially among less-educated and lower-income groups.[28,29]

Compared with previously published studies focusing on socio-demographic differences in fruit and vegetable consumption, the present study has improved the comparability by using standardized data collection methods, including nationally representative data from 21 countries in Europe. When interpreting the results from the present study, several other methodological aspects must be taken into consideration. One of the most important limitation was that only data concerning frequency of fruit and vegetable consumption, rather than portion sizes, were available. Due to limited data, it was not possible to calculate whether the respondents fulfilled the WHO criteria of eating 5 portions of fruit and vegetables per day. Furthermore, the questions used to identify frequency of vegetable consumption did not specify that processed vegetables should be reported as well as consumption of fresh vegetables. As vegetables traditionally are consumed in cooked form in several Eastern European countries, the consumption of vegetables among Eastern European participants may have been underestimated. It should also be noticed that generally, frequency questionnaires often tend to over-estimate the consumption of fruit and vegetables regardless of regional affiliation.[30] The present study may also potentially be biased due to over-reporting intake of fruit and vegetables. Especially those with higher education, and increased knowledge about healthy lifestyle habits, such as eating habits, are often more likely to over-report fruit and vegetable consumption.[31]

## Conclusions

Being female and having high educational level was positively associated with fruit and vegetable consumption in most European regions. However, the results showed some variations in consumption according to educational level and regional affiliation. Knowledge about inequalities in fruit and vegetable consumption according to gender, educational attainment and regional affiliation should inform the development of public health policies and practices across Europe.
